# Lockdown Efficacy in Controlling the Spread of COVID-19 May Be Waning Due to Decline in Public Compliance, Especially among Unvaccinated Individuals: A Cross-Sectional Study in Israel

**DOI:** 10.3390/ijerph19094943

**Published:** 2022-04-19

**Authors:** Moran Bodas, Bruria Adini, Eli Jaffe, Arielle Kaim, Kobi Peleg

**Affiliations:** 1 Department of Emergency & Disaster Management, School of Public Health, Sackler Faculty of Medicine, Tel-Aviv University, P.O. Box 39040, Tel Aviv 6139001, Israel; adini@netvision.net.il (B.A.); ariellekaim@mail.tau.ac.il (A.K.); kobi.peleg@gmail.com (K.P.); 2 Israel National Center for Trauma & Emergency Medicine Research, The Gertner Institute for Epidemiology and Health Policy Research, Sheba Medical Center, Tel Hashomer, Ramat-Gan 5266202, Israel; 3 Public Relations, Training and Volunteers Division, Magen David Adom, Igal Alon 70, Tel Aviv 6706215, Israel; eliy@mda.org.il

**Keywords:** vaccine uptake, lockdown, health regulation, compliance, COVID-19

## Abstract

Public compliance is paramount for the success of public health measures and decision making, such as lockdowns, in controlling the spread of diseases. The aim of this population-based cross-sectional study was to investigate the level of reported compliance with home isolation among the adult Israeli population (*n* = 940) during the first three national lockdowns, compliance with a potential fourth national lockdown if enacted, risk perception of COVID-19, vaccination uptake status, perceived effectiveness of the vaccine, and compliance with additional protective health behaviors (e.g., mask wearing and social distancing). Following widespread compliance with initial lockdowns (90.7% reported “high” or “very high” compliance), as few as 60.1% of participants indicated that they would comply with a fourth lockdown if the government decides to enact it. Non-vaccinated individuals reported the lowest levels of compliance with previous lockdowns, compared to participants who received one or two vaccines and participants vaccinated with three doses. Adjusted for gender and age, the results suggest that fearing being infected with COVID-19, perceiving the vaccine to be effective, and reporting being compliant with other health behaviors—such as mask wearing and maintaining social distance from others—are predictors of lockdown compliance. Considering the effect of pandemic lockdown fatigue, there is little support for additional lockdowns among the Israeli public, unless dramatic changes occur in the characteristics of the COVID-19 pandemic. Compliance with lockdowns is reduced among individuals who are at higher risk of contracting COVID-19, therefore rendering this non-pharmaceutical intervention even less effective in reducing the spread of the disease.

## 1. Introduction

The novel coronavirus disease 2019 (COVID-19) outbreak originated from Wuhan, Hubei, China in December 2019, and has had a significant impact on the global community by disturbing health, economic, societal, and political systems worldwide, resulting in substantial morbidity and mortality [1]. In an effort to contain the spread of the disease, numerous recommendations for public health safety were issued by the World Health Organization (WHO), including using facial coverings (e.g., masks), maintaining social/physical distance between people (e.g., restriction of gatherings), calls for increased personal hygiene, etc. [2]. On a broader scale, public health authorities also utilized lockdowns to reduce the spread of the disease by shutting down businesses, public venues, workplaces, and even leisure locales (e.g., cinemas, coffee shops, public pools, and gyms), effectively confining people to their homes [3,4,5].

Compliance is paramount for the success of public health measures, such as lockdowns, in controlling the spread of diseases [6,7]. In liberal and democratic states, governments need to rely primarily on voluntary and consensual compliance by the public and, in practice, the ability to ensure adherence to the varied measures is dependent on the public’s acquiescence to and implementation of the decisions [8].

However, as the pandemic shifts into an endemic and chronic disease phase—i.e., an ongoing public health hazard with periods of resurgence—people develop so-called pandemic fatigue, which may result in demotivation to follow recommended protective behaviors [9]. Enduring the pandemic for extended periods of time is expected to discourage people from adhering to recommended protective behaviors [10,11,12,13,14].

In Israel, the government enacted three national lockdowns during March 2020, September 2020, and January 2021, in an effort to contain surges in morbidity. During these lockdowns, by government order, non-essential businesses were closed and schools moved to distance teaching; many non-essential workers were moved to work from home; sanctions included fines by the police (although fines were implemented only after repeated non-adherence); and the government issued an assurance that those who were on leaves of absence would be financially compensated by the government for a temporary period of time [14]. These lockdowns took a hefty economic, social, and political toll on Israeli society and, therefore, showed reduced effectiveness in mitigating the spread of the disease. In turn, this led the newly formed government in Israel to explore alternative measures to a fourth lockdown during the surge of the Delta variant during the summer of 2021. One of these measures was the pioneering decision to encourage the public to be vaccinated with a third dose of the vaccine (i.e., a “booster shot”), despite not being approved by any health regulator in the world at that time. Morbidity numbers show that Israel was able to withstand the fourth wave of COVID-19 resurgence without the enforcement of a national lockdown.

To ensure continued general population compliance with health directives and curtailing of the pandemic, it is essential to obtain a strategic and tactical understanding of what potential directives will be adhered to and where compliance will be lacking. The purpose of this study was to examine public compliance with lockdown measures over the course of the COVID-19 outbreak. In addition, the study explored differences in compliance rates between individuals with different levels of COVID-19 vaccine uptake.

## 2. Materials and Methods

### 2.1. Study Design

The study was conducted during the fourth wave of the pandemic in Israel, in September 2021. A sample of the Israeli population (*N* = 940) was employed to assess compliance during the COVID-19 pandemic (throughout various waves of the pandemic) and potential compliance with home isolation during a fourth national lockdown if enacted. Recruitment of participants to the study was conducted through iPanel—an online internet panel company with over 100,000 members, representing all geographic and demographic sectors of the Israeli population. A stratified sampling method was used, based on data published by the Israeli Central Bureau of Statistics with regard to age, gender, religion, and geographic zones.

### 2.2. Participants

The sample size was determined based on the OpenEpi online calculator [15], requiring 384 respondents. This was calculated based on the size of the Israeli population, amounting to 9 million people, as presented by the Israeli Bureau of Statistics. The study was conducted using a random internet sample of 940 participants who consented to participate voluntarily in the research. To partake in the study, the participants had to confirm their willingness to voluntarily participate in the study. The data were collected anonymously, following approval of the Ethics Committee of Tel Aviv University (number 0003846-1 from 2 September 2021).

### 2.3. The Study Tool

The study was based on a structured questionnaire that included items and indices that were developed specifically for this study, given that no prior work had evaluated vaccine uptake for a third dose, as was the case in Israel. The newly developed components of the questionnaire were designed based on a literature review, as well as consultation with experts in the field of behavior of civil society.

### 2.4. Levels of Compliance with Home Isolation

The components of the questionnaire consisted of the following elements: (1) Three items assessing levels of compliance with home isolation (“Extent to which you follow the instructions to stay at home during previous lockdown of (a) first lockdown (March 2020), (b) the second lockdown (October 2020), (c) the third lockdown (December 2020)”) by a 5-point Likert scale, scaling from 1 = to a very small extent, to 5 = to a great extent. (2) One item assessing compliance with a hypothetical fourth lockdown (“And should it be decided to operate a fourth lockdown, to what extent will you be careful to comply with the guidelines?”) by a 5-point Likert scale, scaling from 1 = to a very small extent, to 5 = to a great extent.

### 2.5. Levels of Compliance with Protective Measures (Social Distancing and Mask Wearing)

Two items assessing levels of compliance with the protective measures of social distancing and mask wearing in enclosed spaces (“Given the observed increase in the number of infected with COVID-19 among vaccinated and unvaccinated, to what extent are you now taking care to maintain the following steps compared to the previous waves of the outbreak”) were evaluated by a 5-point Likert scale, scaling from 1 = much more careful, to 5 = much less careful.

### 2.6. Vaccination/Illness Status

One item assessing vaccination status (“Have you been vaccinated against corona?”) was evaluated by a multiple-choice question with the following possible answers: (a) Yes, I got three shots, (b) Yes, I got two shots, (c) Yes, I got one shot, (d) I set a date for a vaccine, or (e) I was not vaccinated. One item assessed the respondents’ history of diagnosis with COVID-19 (Yes/No).

### 2.7. Attitudes towards COVID-19/Vaccine

One item assessing concern regarding the coronavirus (“To what extent are you concerned about the corona virus outbreak?”) was measured on a 5-point Likert scale, from 0 = not at all, to 5 = to a very large extent. One item assessing apprehension towards getting infected with the coronavirus (“How afraid are you to be infected with the corona virus?”) was measured on a 5-point Likert scale, from 1 = to a very small extent, to 5 = to a very large extent. Four items assessing perceived personal threat from the economic, health, security, and political situation in Israel (“In the current situation, how would you rate each of the following situations as threatening to you personally?”) were measured on a 5-point Likert scale, from 1 = not at all threatening, to 5 = threatening to a very large extent.

Two items assessing the perceived importance of the COVID-19 vaccine. (“I believe it is important that I be vaccinated against the corona virus in order to preserve my well-being, the well-being of my dear ones and the well-being of others in society” and “I believe it is important that I get vaccinated against the corona virus because my family members (or close acquaintances) have been vaccinated or intend to get vaccinated”) were measured on a 5-point Likert scale, ranging from 1 = do not agree at all, to 5 = agree very much. Trust in the vaccine was assessed by one item (“I believe in the published information about the effectiveness and safety of the corona vaccine”), on a 5-point Likert scale, ranging from 1 = do not agree at all, to 5 = agree very much.

### 2.8. Attitudes towards Authorities

One item assessing trust in four organizational authorities (the National Ambulance Service, the Health Fund, the Ministry of Health, and the Home Front Command; “To what extent do you trust the following authorities regarding coping with the corona virus”) was measured on a 5-point Likert scale, from 1 = do not trust at all, to 5 = trust very much.

### 2.9. Demographics

Demographics were assessed by 10 items, including gender, year of birth, place of residence, marital status, number of children, number of dependents, education, religion, degree of religiosity, and income.

### 2.10. Statistical Analysis

Descriptive statistics were used to analyze the characteristics of the sample. Pearson correlation coefficients were used for analyzing the associations between the variables that impact on home-isolation compliance. Chi-squared tests were used to evaluate differences between groups by vaccination status (three doses, two doses, or unvaccinated). Participants who received a single dose (*n* = 47, 5%) were excluded from comparative analysis, since most of them (*n* = 40, 85%) were vaccinated with a single dose due to having COVID-19. Independent samples *t*-tests were used to compare means between groups. A linear regression analysis was used to predict compliance with health regulations—specifically lockdowns. All statistical analyses were performed using IBM SPSS software version 27 (IBM Corporation, Armonk, NY, USA); *p*-values lower than 0.05 were considered to be statistically significant.

## 3. Results

A total of 940 participants was included in the sample, of whom 50.6% (*n* = 476) were females. The mean age of the participants was 40.41 years (14.47 SD). Of the total, 137 (14.6%) reported having been diagnosed with COVID-19. The sample included 453 (48.2%) participants who received three doses of the Pfizer (BioNTech) vaccine, 327 (34.8%) who received two doses, 47 (5.0%) who received a single dose, and 113 (12.0%) unvaccinated participants. The complete sociodemographic breakdown of the sample is provided in Table 1. For improved interpretation of sociodemographic data, according to the 2020 census data from the Israeli Central Bureau of Statistics, of Israel’s approximately 9.3 million population, 73.9% are Jewish, roughly 18% are Muslim, 2% are Druze, and 2% are Christian. Approximately 50.23% of the Israeli population is female. Of Israeli Jews over the age of 20 in 2020, 43% self-identify as secular, 22% as traditional but not very religious, 13% as traditional religious, 11% as religious, and 10% as ultra-Orthodox. Regarding education, data from the OECD indicate that over 50.9% of Israelis have a higher education degree [16].

Overall, a majority of participants indicated that they had complied with lockdown regulations when enacted by the government for the last three lockdowns. Nevertheless, for the majority, compliance reduced from the first lockdown (90.7% reported “high” or “very high” compliance), through the second (83.7%), to the third lockdown (69.8%). As few as 60.1% of participants indicated that they would comply with a fourth lockdown if the government decides to impose one.

Reported compliance varied among the participants with the different levels of vaccine uptake, albeit with similar overall trends between lockdowns. Non-vaccinated individuals reported the lowest levels of compliance with previous lockdowns (ranging from 49.6% to 76.1%), compared to participants who received one or two vaccines (65.2% to 91.2%) and participants vaccinated with three doses (78.8% to 94.0%) (see Table 2).

Similar compliance rates and trends were observed when participants were asked about a possible fourth lockdown (see Table 3).

For each participant, a compliance with lockdown index was generated by averaging their responses to the four lockdown-related items. The univariate analysis suggested that this index is associated with age (r(940) = 0.253), being concerned regarding COVID-19 (r = 0.256), fearing contagion with COVID-19 (r = 0.289), perceiving the COVID-19 pandemic as a health threat (r = 0.159), trusting authorities (r = 0.262), trusting the vaccine (r = 0.148), perceiving the vaccine as important (r = 0.329), and reported compliance with other protective behaviors, such as wearing masks and maintaining social/physical distance (r = 0.394), all at a *p*-value of <0.001. In addition, compliance with the lockdown differed between the genders, with females reporting higher willingness to comply with lockdowns (4.14 ± 0.83 SD) compared to males (4.00 ± 0.90 SD), according to independent t-tests (t = 2.475, df = 938, *p* = 0.014). Secular participants reported higher willingness to comply with lockdowns (4.19 ± 0.78 SD) compared to religious participants (3.99 ± 0.92 SD) (t = −3.523, df = 898.3, *p* = 0.001). Participants who were parents to children reported higher willingness to comply with lockdowns (4.14 ± 0.87 SD) than participants without children (3.96 ± 0.84 SD) (t = −3.105, df = 938, *p* = 0.002). Participants with an academic education reported higher willingness to comply with lockdowns (4.18 ± 0.80 SD) than participants without an academic education (3.98 ± 0.92 SD) (t = −3.513, df = 933.6, *p* < 0.001). Lastly, participants who had contracted COVID-19 in the past reported lower compliance with lockdowns (3.85 ± 0.98 SD) than participants who had not been infected with COVID-19 (4.11 ± 0.84 SD) (t = 2.932, df = 172.3, *p* = 0.001).

Based on the bivariate analysis results, a linear regression analysis to investigate compliance with lockdowns was conducted (Table 4). The predictor variables from the univariate analysis were tested a priori to verify that there was no multicollinearity. The predictor variables were entered into the regression model in two blocks (one for demographics and one for attitudinal factors). The results of the analysis suggest that the first model (demographics) explains 34.7% and the second model (demographics + attitudes) explains 51.8% of the total variance of the dependent variable. Adjusted for gender and age, the results suggest that age is a predictor of compliance with lockdowns (β = 0.125). Similarly, fearing being infected with COVID-19 (β = 0.137), perceiving the vaccine to be effective (β = 0.120) and reporting being compliant with other health behaviors—such as mask wearing and maintaining physical distance from others (β = 0.269)—are predictors of lockdown compliance. Lastly, level of vaccine uptake is also a predictor of lockdown compliance (β = 0.082).

## 4. Discussion

The results of this study suggest that compliance with lockdowns is reducing over time. Although a majority of participants indicated complying with past lockdowns and willingness to comply with future lockdowns, their numbers are declining. The first lockdown was met with extensive civilian discipline, and was therefore highly effective in mitigating the spread of the initial COVID-19 wave in Israel [17], China [18], Hungary [19], India [20,21], France [22], Lebanon [23], Portugal [24], and other countries [25,26,27]. However, as COVID-19 progressed, this compliance dissipated gradually because of lockdown fatigue [27]. Moreover, the knowledge that a large part of the population is immunized may increase the Peltzman effect, causing a false sense of security and immunity [28].

In Israel, at the time of this study, only 60% of Israelis indicated their willingness to comply with a possible fourth lockdown. Goldstein, Yeyati, and Sartorio (2021) concluded in the final remarks of their analysis of lockdown efficacy in 152 countries that “heavy reliance on lockdowns that characterized the early stages of the pandemic should be qualified moving forward” [27].

Rendering lockdowns even more ineffective are the results of this study that suggest that compliance is negatively associated with level of individual risk from COVID-19. In other words, unvaccinated individuals, who are at the highest risk of contracting COVID-19, are the most reluctant to comply with health regulations, including lockdowns. Individuals with high risk profiles for contracting COVID-19 seem to be the most prone to taking risks when it comes to protective behavior against COVID-19 [29]. Our findings add to this by suggesting that compliance with COVID-19 health regulations is largely trait-based—either you are highly compliant with most or all health regulations, or you are mostly not. A paradox emerges where those least at risk are the most compliant with health regulations, and vice versa. Under these circumstances, little justification can be found to support additional national lockdowns.

Instead, governments may want to adopt policies that benefit individuals who actively participate in reducing the risk of the spread of COVID-19, e.g., through vaccination or regular testing. The “green pass” approach developed in Israel is one example for incentivizing the public to be vaccinated by offering fully vaccinated and COVID-19-recovered individuals access to social, cultural, and leisure activities, as well as exemption from self-quarantine after coming into contact with a confirmed COVID-19 case [30]. The challenges presented by “green passes” or, alternatively put, “vaccination passports”, are new but mostly familiar. While attempts to avoid discrimination and inequity must be made to ensure free choice and fair use, this strategy to increase vaccine uptake and reduce the overall threat posed by COVID-19 is of value [31].

Another important finding of this study relates to public trust. Although previous studies have demonstrated the importance of public trust in authorities for public compliance with health regulations [7,32], the results of the present study suggest that trust in authorities has little-to-no effect on individual compliance with health regulations. This finding is similar to those reported elsewhere [33,34]. Nevertheless, the results of this study also suggest that belief in the vaccine’s efficacy—and especially perceiving it as important—does play a major role in public compliance with health regulations—especially lockdowns. Further examination of the relationship between trust and compliance with health regulations during the ongoing pandemic is warranted in order to elucidate a clearer picture of the situation.

A study by Liat Ayalon (2021) that looked specifically into the trust profile of Israelis during the COVID-19 outbreak elucidated the important distinction between trust in the government and trust in science and medical professionals. According to the author, the most likely to comply with health regulations were individuals presenting high trust in the latter [28]. Similar findings have been reported elsewhere [35,36]. The results of this study provide additional support for this assertion. As COVID-19 progresses into a chronic threat, those most likely to comply with health regulations are those convinced by scientists and medical doctors about the importance, efficacy, and safety of vaccination. Perhaps finding comfort and stability in science may suffice in bringing down rebellious attitudes toward government strategies in combating the spread of COVID-19.

Alternative approaches to promoting public compliance with health regulations during pandemics are required. For instance, Kuiper et al. (2020) reported that government lockdown approaches were successful in the Netherlands primarily because they relied on moral appeals and self-discipline. According to the authors, “compliance was lower for people who lacked the practical capacity to follow the measures and for those who have the opportunity to break the measures” [37].

In particular, the results highlight the importance of adapting and adjusting current risk communication efforts [38]. Instead of focusing on fear appeal tactics, which are already known to be mostly counterproductive in promoting health behaviors [39,40], risk communication in the age of the ongoing threat of COVID-19 should emphasize normative behaviors that value protective behavior not just for oneself, but also for others [41,42]. This risk communication should empower people to assume personal responsibility and increase their trust in science and medicine by providing evidence in a clear and straightforward manner [36].

This study has several limitations. First, although efforts were made to make the tool reliable through expert consultations, this study employs the use of non-validated tools that were designed for the purpose of this research. Second, this study utilized an online panel to collect responses. While this option provides immediate access to a diverse sample of the population across a wide geographic distribution, it may limit the generalization of the conclusions to people with high digital literacy. Third, although this study was able to capture a sizeable portion of unvaccinated participants, it is difficult to assess whether or not these participants are representative of this group. Fourth, this study was performed in Israel; generalization of the conclusions to other populations should be done with caution.

## 5. Conclusions

We argue that given the current pharmaceutical interventions (i.e., vaccines), and especially when considering the effect of pandemic lockdown fatigue, there is little support for additional lockdowns unless dramatic changes occur in the characteristics of the COVID-19 pandemic. Moreover, our results show that compliance with lockdowns is reduced among individuals who are at higher risk of contracting COVID-19, therefore rendering this non-pharmaceutical intervention even less effective in reducing the spread of the disease. Public intent to comply with health regulations is a crucial component in public health policy. Alternative approaches to promoting public compliance with health regulations should be considered.

## Figures and Tables

**Table 1:** Sociodemographic distribution of the studied sample.

Variable	*N* (%)
**Gender**	
Female	476 (50.6%)
Male	464 (49.4%)
**Age (mean 40.41, SD 14.47)**	
18–35	411 (43.7%)
36–55	340 (36.2%)
56–70	189 (20.1%)
**Religion**	
Jewish	746 (79.4%)
Muslim	122 (13.0%)
Druze	29 (3.1%)
Christian	36 (3.8%)
Other	7 (0.7%)
**Religiosity**	
Secular	384 (40.9%)
Traditional	350 (37.2%)
Religious	120 (12.8%)
Ultra-Orthodox	81 (8.6%)
Other	5 (0.5%)
**Place of residence**	
Haifa and North	333 (35.4%)
Tel Aviv and Center	249 (26.5%)
South and Coastline Plain	195 (20.7%)
Greater Jerusalem	78 (8.3%)
HaSharon Region	85 (9.0%)
**Family status**	
Coupled with children	534 (56.8%)
Coupled without children	143 (15.2%)
Single with children	69 (7.3%)
Single without children	194 (20.6%)
**Children (under 18 years)**	
Yes	422 (44.8%)
No	181 (19.3%)
Missing	337 (35.9%)
**Education**	
Up to (including) 8 years	12 (1.3%)
Up to (including) 12 years	280 (29.8%)
Vocational degree	194 (20.6%)
Academic degree	454 (48.3%)
**Income**	
Much below average	295 (31.4%)
Below average	200 (21.3%)
Average	199 (21.2%)
Above average	129 (13.7%)
Much above average	45 (4.8%)
Missing	72 (7.7%)

**Table 2:** Frequency (*N* (%)) of reported compliance with lockdowns according to vaccination status (*N* = 940).

Lockdown	Compliance Level	Vaccination Status			Chi-Squared
		3 Doses	1–2 Doses	Unvaccinated	
1st lockdown (March–May 2020)	Little or very little	11 (2.4%)	11 (2.9%)	13 (11.5%)	37.2 ***
	Medium	16 (3.5%)	22 (5.9%)	14 (12.4%)	
	High or very high	426 (94.0%)	341 (91.2%)	86 (76.1%)	
2nd lockdown (September–October 2020)	Little or very little	12 (2.6%)	18 (4.8%)	16 (14.2%)	48.1 ***
	Medium	36 (7.9%)	46 (12.3%)	25 (22.1%)	
	High or very high	405 (89.4%)	310 (82.9%)	72 (63.7%)	
3rd lockdown (January–February 2021)	Little or very little	26 (5.7%)	46 (12.3%)	30 (26.5%)	57.0 ***
	Medium	70 (15.5%)	84 (22.5%)	27 (23.9%)	
	High or very high	357 (78.8%)	244 (65.2%)	56 (49.6%)	

*** *p* < 0.001.

**Table 3:** Distribution (*N* (%)) of public attitudes toward a possible fourth lockdown according to vaccination status (*N* = 940).

Lockdown	Compliance Level	Vaccination Status			Chi-Squared
		3 Doses	1–2 Doses	Unvaccinated	
Compliance with 4th lockdown if enacted	Little or very little	65 (14.3%)	91 (24.3%)	44 (38.9%)	51.4 ***
	Medium	70 (15.5%)	80 (21.4%)	25 (22.1%)	
	High or very high	318 (70.2%)	203 (54.3%)	44 (38.9%)	
Perception of 4th lockdown efficacy if enacted	Little or very little	216 (47.7%)	191 (51.1%)	72 (63.7%)	10.1 *
	Medium	116 (25.6%)	87 (23.3%)	23 (20.4%)	
	High or very high	121 (26.7%)	96 (25.7%)	18 (15.9%)	

*** *p* < 0.001, * *p* < 0.05.

**Table 4:** Results of linear regression analysis to predict compliance with lockdowns (*N* = 940).

Model		Unstandardized Coefficients		Standardized Coefficients	t	F	R-Squared
		B	Std. Error	Beta			
1	(Constant)	4.174	0.134		31.075 ***	17.895 ***	34.7%
	Gender	−0.144	0.054	−0.084	−2.672 **		
	Age	0.010	0.002	0.174	4.505 ***		
	Religiosity	0.077	0.055	0.045	1.407		
	Having children	−0.049	0.066	−0.028	−0.743		
	Education	0.073	0.055	0.043	1.338		
	History of COVID-19 infection	0.050	0.082	0.021	0.614		
	Vaccine uptake	0.297	0.046	0.235	6.523 ***		
2	(Constant)	3.965	0.344		11.533 ***	23.910 ***	51.8%
	Gender	−0.090	0.050	−0.052	−1.793		
	Age	0.007	0.002	0.125	3.516 ***		
	Religiosity	0.078	0.051	0.045	1.537		
	Having children	−0.054	0.060	−0.030	−0.892		
	Education	0.093	0.050	0.054	1.845		
	History of COVID-19 infection	0.019	0.078	0.008	0.244		
	Vaccine uptake	0.103	0.051	0.082	2.020 *		
	Worry about COVID-19	0.034	0.035	0.039	0.988		
	Fear of infection with COVID-19	0.105	0.033	0.137	3.127 **		
	COVID-19 perceived as health threat	0.019	0.028	0.025	0.677		
	Trusting the vaccine	0.002	0.041	0.001	0.041		
	Trusting the authorities	0.082	0.061	0.067	1.358		
	Perceiving the vaccine to be important	0.104	0.043	0.120	2.394 *		
	Compliance with other protective behavior (e.g., mask wearing and social distancing)	0.240	0.027	0.269	8.812 ***		

Regression was performed in Enter mode with two blocks. The first block included demographic variables, while the second block included attitudinal factors concerning the COVID-19 outbreak. Gender: 0 = female, 1 = male; Age (cont.). Religiosity: 0 = religious, 1 = secular. Having children: 0 = no, 1 = yes. Education: 0 = non-academic, 1 = academic. History of COVID-19 infection: 0 = no, 1 = yes. Vaccination uptake: 1 = unvaccinated, 2 = one or two doses, 3 = three doses. Worry about COVID-19 and fear of infection with COVID-19 (ordinal—5-point Likert scale from 1 = very little to 5 = very much). Trusting the vaccine, trusting the authorities, perceiving the vaccine to be important, and compliance with other protective behaviors (all cont.). * results are significant at the 0.05 level (two-tailed), ** at the 0.01 level (two-tailed), and *** at the 0.001 level (two-tailed).

## Data Availability

The data presented in this study are available upon request from the corresponding author.

## References

[1] Kaim A., Gering T., Moshaiov A., Adini B. (2021). Deciphering the COVID-19 Health Economic Dilemma (HED): A Scoping Review. Int. J. Environ. Res. Public Health.

[2] World Health Organization (WHO) (2020). Pandemic Fatigue—Reinvigorating the Public to Prevent COVID-19. https://apps.who.int/iris/bitstream/handle/10665/337574/WHO-EURO-2020-1573-41324-56242-eng.pdf.

[3] Cohen J., Kupferschmidt K. (2020). Countries Test Tactics in ‘War’ against COVID-19. Science.

[4] Pascarella G., Strumia A., Piliego C., Bruno F., Del Buono R., Costa F., Scarlata S., Agrò F.E. (2020). COVID-19 diagnosis and management: A comprehensive review. J. Intern. Med..

[5] Güner H.R., Hasanoğlu I., Aktaş F. (2020). COVID-19: Prevention and control measures in community. Turk. J. Med. Sci..

[6] Sailer M., Stadler M., Botes E., Fischer F., Greiff S. (2021). Science knowledge and trust in medicine affect individuals’ behavior in pandemic crises. Eur. J. Psychol. Educ..

[7] Bodas M., Peleg K. (2020). Self-isolation compliance in the COVID-19 era influenced by compensation: Findings from a recent survey in israel. Health Aff..

[8] Bradford B., Hobson Z., Kyprianides A., Yesberg J., Jackson J., Posch K. (2020). Policing the Lockdown: Compliance, Enforcement and Procedural Justice.

[9] Bodas M., Kaim A., Velan B., Ziv A., Jaffe E., Adini B. (2022). Overcoming the effect of pandemic fatigue on vaccine hesitancy—Will belief in science triumph?. J. Nurs. Scholarsh..

[10] Nivette A., Ribeaud D., Murray A., Steinhoff A., Bechtiger L., Hepp U., Shanahan L., Eisner M. (2020). Non-compliance with COVID-19-related public health measures among young adults in Switzerland: Insights from a longitudinal cohort study. Soc. Sci. Med..

[11] Carlitz R.D., Makhura M.N. (2020). Life under lockdown: Illustrating tradeoffs in South Africa’s response to COVID-19. World Dev..

[12] Denford S., Morton K.S., Lambert H., Zhang J., Smith L.E., Rubin J.G., Cai S., Zhang T., Robin C., Lasseter G. (2020). Understanding patterns of adherence to COVID-19 mitigation measures: A qualitative interview study. medRxiv.

[13] Bodas M., Peleg K. (2021). Pandemic Fatigue: The Effects Of The COVID-19 Crisis On Public Trust And Compliance With Regulations In Israel. Health Aff..

[14] Kaim A., Siman-Tov M., Jaffe E., Adini B. (2021). Factors that enhance or impede compliance of the public with governmental regulation of lockdown during COVID-19 in Israel. Int. J. Disaster Risk Reduct..

[15] Dean A.G., Sullivan K.M., Soe M.M. OpenEpi: Open Source Epidemiologic Statistics for Public Health, Version. Updated 2013/04/06. www.OpenEpi.com.

[16] Weinreb A. (2020). Population projections for Israel, 2017–2040. Policy Pap..

[17] de Bruijn A.L., Feldman Y., Kuiper M.E., Brownlee M., Reinders Folmer C., Kooistra E.B., Olthuis E., Fine A., van Rooij B. (2020). Why did Israelis comply with COVID-19 Mitigation Measures during the initial first wave lockdown?. Amst. Law Sch. Res. Pap..

[18] Lau H., Khosrawipour V., Kocbach P., Mikolajczyk A., Schubert J., Bania J., Khosrawipour T. (2020). The positive impact of lockdown in Wuhan on containing the COVID-19 outbreak in China. J. Travel Med..

[19] Röst G., Bartha F.A., Bogya N., Boldog P., Dénes A., Ferenci T., Horváth K.J., Juhász A., Nagy C., Tekeli T. (2020). Early phase of the COVID-19 outbreak in Hungary and post-lockdown scenarios. Viruses.

[20] Krishan K., Kanchan T. (2020). Lockdown is an effective’vaccine’against COVID-19: A message from India. J. Infect. Dev. Ctries..

[21] Sardar T., Nadim S.S., Rana S., Chattopadhyay J. (2020). Assessment of lockdown effect in some states and overall India: A predictive mathematical study on COVID-19 outbreak. Chaos Solitons Fractals.

[22] Roques L., Klein E.K., Papaïx J., Sar A., Soubeyrand S. (2020). Impact of lockdown on the epidemic dynamics of COVID-19 in France. Front. Med..

[23] Kharroubi S., Saleh F. (2020). Are lockdown measures effective against COVID-19?. Front. Public Health.

[24] Peixoto V.R., Vieira A., Aguiar P., Carvalho C., Thomas D., Abrantes A. (2020). Rapid assessment of the impact of lockdown on the COVID-19 epidemic in Portugal. MedRxiv.

[25] Alfano V., Ercolano S. (2020). The efficacy of lockdown against COVID-19: A cross-country panel analysis. Appl. Health Econ. Health Policy.

[26] Atalan A. (2020). Is the lockdown important to prevent the COVID-19 pandemic? Effects on psychology, environment and economy-perspective. Ann. Med. Surg..

[27] Goldstein P., Yeyati E.L., Sartorio L. (2021). Lockdown fatigue: The diminishing effects of quarantines on the spread of COVID-19. CID Work. Pap. Ser..

[28] Iyengar K.P., Ish P., Botchu R., Jain V.K., Vaishya R. (2021). Influence of the Peltzman effect on the recurrent COVID-19 waves in Europe. Postgrad. Med. J..

[29] Ayalon L. (2021). Trust and compliance with COVID-19 preventive behaviors during the pandemic. Int. J. Environ. Res. Public Health.

[30] Wilf-Miron R., Myers V., Saban M. (2021). Incentivizing vaccination uptake: The “green pass” proposal in Israel. JAMA.

[31] Dye C., Mills M.C. (2021). COVID-19 vaccination passports. Science.

[32] Almutairi A.F., BaniMustafa A.A., Alessa Y.M., Almutairi S.B., Almaleh Y. (2020). Public trust and compliance with the precautionary measures against COVID-19 employed by authorities in Saudi Arabia. Risk Manag. Healthc. Policy.

[33] Clark C., Davila A., Regis M., Kraus S. (2020). Predictors of COVID-19 voluntary compliance behaviors: An international investigation. Glob. Transit..

[34] Wong C.M.L., Jensen O. (2020). The paradox of trust: Perceived risk and public compliance during the COVID-19 pandemic in Singapore. J. Risk Res..

[35] Plohl N., Musil B. (2021). Modeling compliance with COVID-19 prevention guidelines: The critical role of trust in science. Psychol. Health Med..

[36] Bicchieri C., Fatas E., Aldama A., Casas A., Deshpande I., Lauro M., Parilli C., Spohn M., Pereira P., Wen R. (2021). In science we (should) trust: Expectations and compliance across nine countries during the COVID-19 pandemic. PLoS ONE.

[37] Kuiper M.E., de Bruijn A.L., Reinders Folmer C., Olthuis E., Brownlee M., Kooistra E.B., Fine A., Van Rooij B. (2020). The intelligent lockdown: Compliance with COVID-19 mitigation measures in the Netherlands. Amst. Law Sch. Res. Pap..

[38] Warren W.G., Lofstedt R. (2021). Risk Communication and COVID-19 in Europe: Lessons for Future Public Health Crises. J. Risk Res..

[39] Ruiter R.A., Kessels L.T., Peters G.J.Y., Kok G. (2014). Sixty Years of Fear Appeal Research: Current State of the Evidence. Int. J. Psychol..

[40] Kaim A., Siman-Tov M., Jaffe E., Adini B. (2021). From Isolation to Containment: Perceived Fear of Infectivity and Protective Behavioral Changes during the COVID-19 Vaccination Campaign. Int. J. Environ. Res. Public Health.

[41] Sheeran P., Maki A., Montanaro E., Avishai-Yitshak A., Bryan A., Klein W.M., Miles E., Rothman A.J. (2016). The impact of changing attitudes, norms, and self-efficacy on health-related intentions and behavior: A meta-analysis. Health Psychol..

[42] Agranov M., Elliott M., Ortoleva P. (2021). The importance of Social Norms against Strategic Effects: The case of COVID-19 vaccine uptake. Econ. Lett..

